# Aptamer-Based Single-Step Assay by the Fluorescence Enhancement on Electroless Plated Nano Au Substrate

**DOI:** 10.3390/s17092044

**Published:** 2017-09-07

**Authors:** Jegatha Nambi Krishnan, Sang-Hwi Park, Sang Kyung Kim

**Affiliations:** 1 Center for BioMicrosystems, Korea Institute of Science and Technology, 39-1 Hawolgok-dong, Sungbuk-gu, Seoul 136-791, Korea; jegathak@goa.bits-pilani.ac.in (J.N.K.); kunchs@hanmail.net (S.-H.P.); 2 School of Micro Nano System Engineering, Korea University of Science and Technology, Daejeon 305-333, Korea; 3 School of Chemical Engineering, Birla Institute of Technology and Science, Pilani, K. K. Birla Goa Campus, Zuari Nagar, Goa 403-726, India

**Keywords:** aptamer, electroless deposition, gold nanostructure, thrombin, human serum albumin, fluorescence, optical biosensor

## Abstract

A new single-step aptamer-based surface-enhanced fluorescent optical sensor is built, by combining an aptamer–target interaction for target recognition and a fluorophore interaction for signal enhancement. The developed aptasensor is simple, sensitive, specific and stable for the detection of thrombin. A new nanometallic Au structure in the range of 100 nm was constructed through effective electroless plating method on a Cu thin film. Cu^+^ ions act as sacrificial seeds for the reduction of Au^2+/3+^ ions to form Au nanolawns. In order to utilize the structure for a fluorescence-based sensor, aptamer conjugated with Cy3 was immobilized on the nanogold substrate through electrostatic attraction. The Au substrate was coated with chitosan (molecular weight 1000 Da). Thrombin binding aptamer (TBA) was applied as a model system demonstrating the aptamer-based fluorescence assay on nanogold substrates. Thrice-enhanced fluorescence emission was achieved with Cy3-conjugated TBA stably immobilized on the chitosan-coated Au substrate. The intensity change was proportional to the concentration of thrombin from 10 μM to 10 pM, whereas the intensity change was ignorable for other proteins such as human serum albumin (HSA). Aptamer-based assay benefited from simple immobilization of receptors and Au nanostructure contributed in building an effective surface enhancing/positively charged substrate was proved. Such an aptasensor holding high utilities for point-of-care devices by incorporating simplicity, sensitivity and selectivity in detection, low-cost for test, small sample volumes has been developed.

## 1. Introduction

Detection and tracking of proteins and other small molecules is of high priority not only in the field of medical diagnostics and drug development, but also in biological and biotechnology research, environmental monitoring, forensic investigations, and biodefense. Recently, researchers of different expertise develop methodologies/techniques that are sensitive, specific, robust, high-throughput, simple, and cost-effective. Most of the methods require molecular recognition events such as formation of antibody–antigen or aptamer–analyte complexes as in “sandwich type” enzyme-linked immunosorbent assays (ELISA) to detect biomolecules while the use of receptors tagged with probe molecules is undesirable [1,2,3]. More recently, aptamers which are functional single-stranded oligonucleotides (DNA or RNA) generated by the process called systematic evolution of ligands by exponential enrichment (SELEX) [4] have been a growing focus of many scientists. They possess all advantages of antibodies and unique merits such as thermal stability. They can be obtained ex vivo and it can be produced in large scale at low cost. They can bind to their target molecules selectively and with high affinity by forming secondary structures and shapes (e.g., quadruplex, hairpin loop, and T-junction) [5,6]. Aptamers have been used successfully in many applications, including flow cytometry; [7], biosensors [8], ELISA type assays [9] and separations, such as capillary electrochromatography and affinity chromatography [10]. Several aptamers have been developed for the detection of certain biological and chemical threat agents [e.g., trinitrotoluene (TNT) [11]; cancer biomarkers [12]; and other biologically important biomolecules such as thrombin [13].

Thrombin is a specific serine protease involved in conversion of soluble fibrinogen into insoluble strands of fibrin. It is crucial in physiological and pathological coagulation, and regulates many processes in inflammation and tissue repair at the vessel wall [14]. The concentration of thrombin in blood during the coagulation progress varies from nM to low μM levels [15] while the detection at high pM range is important for related diagnoses [16]. Therefore, it is necessary to develop a sensor towards thrombin detection with high sensitivity and selectivity.

For simple assays, electrochemical [17,18] and optical techniques [19] were widely applied in the field of thrombin detection with various methods. There are approaches that make use of the advantage of molecular beacons in which conformational change of aptamer registers the protein–aptamer recognition process and induces fluorescence signal variation [20]. However, such approaches require dual-label on the aptamer beacon. Label-free target protein can be detected through the structure-induced fluorescence enhancement of guanine-quenching aptamer with two major advantages that only one fluorophore is attached and the target protein is not labeled, which maintains the structure and activity of protein leading to a cost-effective sensor with simple assay protocol [20]. However, the detection limit of both the hairpin design and the duplex design was affected by buffer solution. The search for an appropriate buffer is an issue that should be considered seriously when establishing a protocol of aptamer-based assay.

There are several reports on fluorescence enhancement from an array of Au nanoparticles. Fluorescently labeled aptamers were displaced from Au nanoparticles when target proteins construct complex with aptamers [21,22]. The advantages of simplicity and specificity, the use of aptamer gold nanoparticles may be well suited for protein analysis and cancer diagnosis. Those highly sensitive aptasensors based on surface plasma resonance [21], surface-enhanced resonance Raman scattering [23] and a graphene field-emit transistor require elaborate process [22] for sensor fabrication which limited their extended applications. Moreover, it is significant to suppress the nonspecific adsorption of proteins and DNA by modifying the surface with biocompatible polymers such as poly(N-vinyl-2-pyrrolidone) [24].

In addition, metal-induced fluorescence quenching arises primarily from nonradiative energy transfer from the dye to the metal. Strouse and co-workers further revealed a novel nanosurface energy-transfer effect occurring at the surface of gold nanoparticles (AuNPs), which leads to long-range energy-transfer-based fluorescence quenching. Based on this superquenching effect, AuNPs have been successfully employed to develop nanosensors for sensitive DNA detection [25,26]; importantly given that AuNPs can efficiently quench almost all fluorophores. However, one of the important applications of surface plasmon is the enhancement of dye fluorescence. Fluorescence from a single-dye molecule adsorbed on a spheroidal metal particle has been theoretically studied by Gersten and Nitzan. They found that the enhancement of fluorescence intensity strongly depends on the quantum yield of the dye molecule, the distance from the surface to the dye, and the size and shape of the particle. These theoretical predictions about the quantum yield and the distance have been experimentally confirmed [27,28]. The influence of particle size on fluorescence enhancement was reported by Nakamura using Rose Bengal on spherical gold nanoparticles. The enhancement of fluorescence intensity would strongly depend on the quantum yield of dye molecule, distance from the surface to dye and size, and shape of particle [29,30]. Oroval et al. reported the preparation of aptamer-gate silica mesoporous supports for the selective and sensitive fluorogenic signaling of α-thrombin [31].

**Figure 1:**
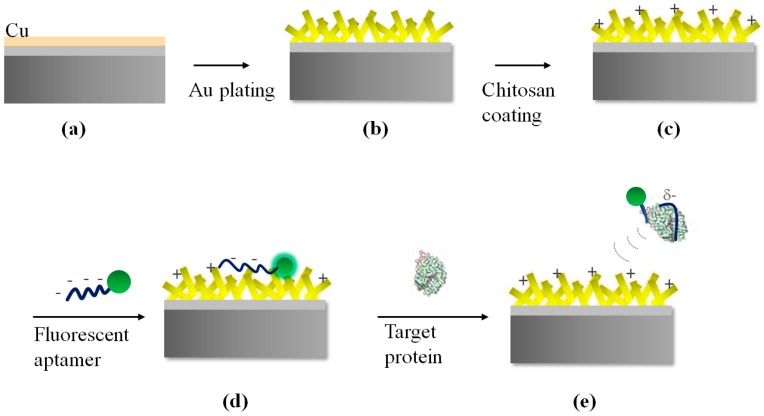
Schematic of aptamer-based fluorescence assay on fabricated Au nanostructures. (**a**) Cu deposited by e-beam evaporation; (**b**) Au nanostructures fabricated through electroless plating; (**c**) Chitosan-coated Au nanostructures; (**d**) Binding of fluorescent DNA to Au nanostructures; (**e**) specific aptamer–target binding leaving behind the chitosan-coated Au nanostructures.

In this paper, gold nanostructures were accomplished from quick electroless plating of Cu thin film. The proposed procedure was demonstrated with high sensitivity and selectivity reducing the requirement of large sample volumes, expensive reagents or multiple steps associated with incubation and washing. In this paper, a convenient new aptasensor fabrication (Figure 1) and reliable detection platform for thrombin is developed on the basis of electrostatic interaction. Both electrostatic interaction between the positively charged polymer chitosan and negatively charged thrombin aptamer and the fluorescence enhancement of adsorbed to the gold nanostructures were involved in the procedure to enable high sensitivity and selectivity.

## 2. Materials and Methods

### 2.1. Fabrication of Substrates with Au Nanostructure

The crystalline (100) p-type Si wafers were cleaned by standard RCA method before any process, and then each Si surface was deposited with Ti 100 Å. The metal Cu of thickness 1000 Å was deposited onto a Ti layer of 100 Å used as an adhesive layer between Si substrate and the metallic layer. Both Ti (100 Å) and metallic layer (1000 Å) were deposited using an e-beam evaporator (Ultech, Daegu, Korea) at a rate of 3 Å/s. The evaporation base pressure was 2 × 10^−6^ Torr. After metal deposition, the substrate was immersed into the electroless plating solution which contained dilute Au metal salt [KAu(CN)_2_] (CnCTech, Chandler, AZ, USA) in the range of 0.2–0.4% (wt.). The catalytic reduction process of gold ions in this aqueous solution results in the subsequent deposition of gold without the use of electrical energy. The process temperature of the solution was maintained at 85 °C using a double boiler. The replacement time was 2 min. The processing temperature and pH were monitored using a (ELMETRON, CPC-401, Zabrze, Poland). The solution was stirred with a magnetic stirrer. The stirring speed of the magnetic stirrer was maintained at 50 rpm.

### 2.2. Chitosan Coating Process

The chitosans with different molecular weight (<1000, 1000–3000, >5000) were purchased from Kittolife (Seoul, Korea). The chitosan powder of 220 mg was mixed in 1.5 mL of acetic acid. The fabricated gold nanostructures were immersed in the prepared chitosan solution for 4 h, after which the chitosan solution was just removed from the substrate. The substrates were immersed in deionized water for further removal of excess chitosan. The washing condition was optimized to immersion for 30 min with shaking at 280 rpm. The morphology of the Au nanostructure was observed with scanning electron microscopy (SEM: FEI Company, Nova NanoSEM 200, Hillsboro, OR, USA) before and after chitosan coating.

### 2.3. Immobilization of Aptamers and Optical Signal Measurement

Thrombin binding aptamer (10 μM, Bionics, Seoul, Korea) and the binding buffer (20 mM MgCl_2_, 20 mM Tris-HCl, pH 7.4) were chosen for this study. Below are the three aptamer DNAs used, namely:

Thrombin binding aptamer: CAC TGT GGT TGG TGT GGT TGG (21mer)-Cy5 (or FAM);
Thrombin binding aptamer: GGT TGG TGT GGT TGG (15mer)-Cy5 (or FAM);
Random DNA sequence: TAG TGA ATG GAT CGG ACA GC (20mer)-Cy5 (or FAM).

Underlined sequence is thrombin binding area. The aptamer DNA was purchased from Bionics, Korea, while thrombin was purchased from Sigma Aldrich (St. Louis, MO, USA).

The substrate is ready for adsorption of fluorescent DNA and DNA was dissolved in the binding buffer to the concentration of 1 μM. A droplet of DNA solution (20 μL) was added on the substrate for 1 h incubation. Then, partially adhered DNAs were removed after 30 min rinsing with deionized water with agitation. Then, the DNA tethered substrates were treated with 1% bovine serum albumin (BSA) (Sigma Aldrich, St. Louis, MO, USA) in the binding buffer for 10 min and rinsed with deionized water.

The experiment was performed for DNA conjugated with dye, Cy3 and FAM, adsorbed on gold nanostructures. These results suggest that the control of chitosan coating is especially important to realize an efficient enhancement of dye fluorescence. Fluorescence images were obtained from laser scanning microscope 5 Pascal (Zeiss, Oberkochen, Germany) and the intensity was quantified with Scanarray Express (Perkin Elmer, Waltham, MA, USA). The excitation source was the 488 nm and for the measurements of fluorescence excitation, 510–550 nm filter was integrated and the exposure time was set to 5 ms.

## 3. Results and Discussion

### 3.1. Fabrication of Gold Nanostructure Substrate for Aptasensor

The galvanic displacement process [32] was used to achieve gold nanostructure substrate for aptasensors. The galvanic displacement is a plating process in which metal ions in plating solution displace a metal atom on the surface of a substrate metal by a strong redox displacement reaction. This plating process is advantageous in forming a uniform thickness and is simple. The catalytic reduction process of gold ions in this aqueous solution results in the subsequent deposition of gold metal without the use of electrical energy. Figure 1a,b depicts the images of Cu deposited by e-beam evaporation and gold nanostructures fabricated through electroless plating. The immersion plating process occurs when there is a difference in ionization tendency of metals present on top of the Si substrate and that present in the solution. At first step of reaction, gold grains are distributed as seeds on the metallic substrates. Thereafter, the reaction vessel containing the Au plating solution is removed from the hot plate. The Au plating solution containing the substrates was allowed to cool at room temperature. The copper substrate proved to be fast and reliable in the fabrication of anisotropic nanostructures for surface enhancement among various metal substrates used for the growth of Au nanostructures. Moreover, copper target is cost-effective for thin-film deposition achieved through e-beam evaporation.

**Figure 2:**
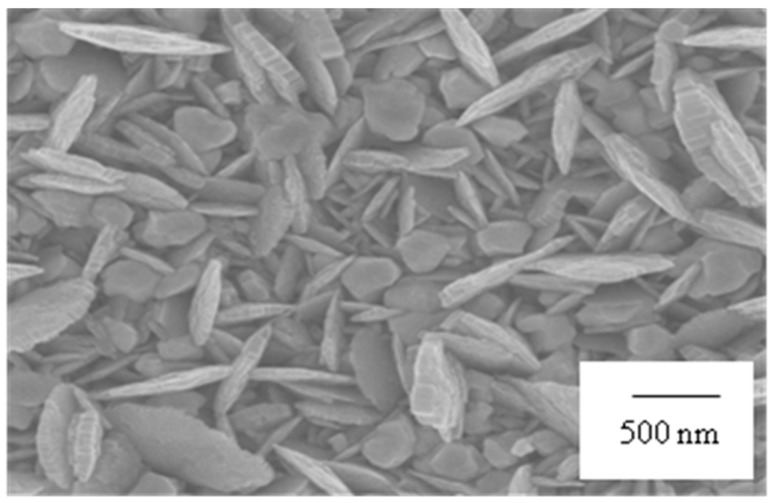
SEM image of Au nanostructures formed on the copper substrate after 4 min electroless plating at 85 °C.

It is well understood that the details of Au nanostructures are related to several other reaction parameters as well as the type of substrate metals. Therefore, effect of physical parameters such as temperature, time and convection was evaluated to set the optimum condition for uniform growth of anisotropic Au nanostructure. By maintaining 2 min as seeding time of plating, the temperature for seeding step was varied from 75 °C to 90 °C. The samples were collected at different seeding temperatures of 75 °C, 80 °C, 85 °C and 90 °C. The structural morphology of each sample was observed to be unique through SEM characterization. After the seeding step, Au was plated under natural cooling of solution for 2 min. In order to study the controlling parameters on nanostructure formation, the incubation time was varied from 2 min to 24 h. It was found that there is no remarkable change in the structural morphology of Au nanostructure with increased incubation time of over 2 min. This elucidates that the growth of nanostructure was achieved within 2 min of gradual cooling period. On the contrary, rapid cooling in no seconds resulted in spherical Au nanostructures. Thus, temperature is evident to be one of the most critical parameters in nanostructure growth. The sharp-edged nanolawn structure was achieved at seeding temperature of 85 °C and natural cooling time of 2 min. As mass transport affected the uniformity of the film, mild agitation at 50 rpm provided reliable fabrication of Au nanostructures as a film. In the same way, the effect of Au ion concentration on the growth of anisotropic Au nanostructures was also investigated. By lowering the concentration of Au ion to 0.2%, more advantageous sharp nanolawn morphology of Au nanostructures was obtained. The optimum plating condition to achieve 200–250 nm of sharp and uniform gold nanostructures was found to be 85 °C, and 2 min followed by gradual cooling stirring at 50 rpm with Au ion concentration of 0.2%. Figure 2 shows the Au nanostructures grown on Cu substrates through 4 min electroless plating under seeding condition at 85 °C.

### 3.2. Positive Layer Polymer

**Figure 3:**
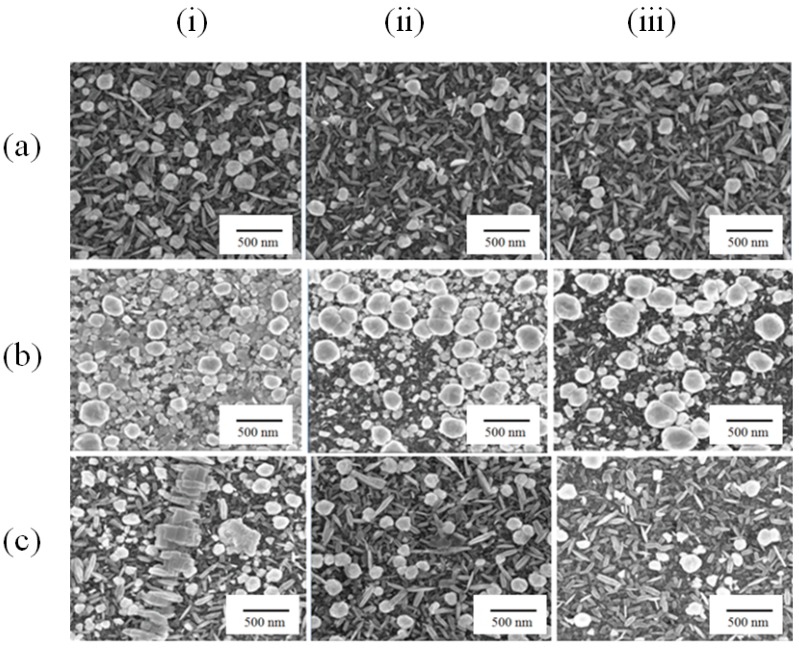
SEM images of surface treatment of Au nanostructures with chitosan coating. Chitosan of (**a**) 1000 Da (**b**) 1000–3000 Da and (**c**) 5000 Da coated Au nanostructures (**i**) only immersion for 30 min (**ii**) immersion with shaking for 15 min and (**iii**) immersion with shaking for 30 min.

The fabricated gold nanostructures are coated with positively charged polymer to locate DNA-based aptamers utilizing their strong negative charge density (Figure 1c). Chitosan was coated to develop positive charges on the Au nanostructures and provide strong electrostatic attraction to DNAs. However, thick layer of polymer could reduce the effect of Au nanostructure on signal enhancement. In order to optimize the chitosan coating, chitosan of different molecular weights such as 1000, 1000–3000 and 5000 Da were chosen for study. The substrates were immersed in chitosan solution for 4 h and then, rinsed with DI water under rigorous agitation to remove any excess amount. From Figure 3a–c, it is clear that chitosan layers were thinned gradually with respect to washing conditions. Chitosan with different molecular weights generated unique structure of chitosan ~100 nm on the substrate of Au nanostructure. The mixed chitosan of molecular weights 1000–3000 precipitated in the most irregular manners and was hardly removed from rinsing process. In the case of chitosan 1000 and 5000 Da, chitosan structures were successfully removed after 30 min rinsing with deionized water. The chitosan of 1000 and 5000 Da were used on the Au nanostructure for further development of sensor.

After the substrate is coated with a thin layer of chitosan, a droplet of DNA aptamer solution was added onto the substrate for 1 h incubation. The 3’ end of each DNA aptamer was linked to Cy3 moiety as shown in Figure 1d. Then, weakly adhered aptamers were removed after 30 min rinsing with deionized water and agitation (Figure 1d). The same is verified with FAM-conjugated DNA aptamers. Then, the substrates tethered with fluorescent aptamers were treated with BSA to reduce non-specific response of the aptamers from other proteins, perturbing the charge environment of chitosan and DNA.

**Figure 4:**
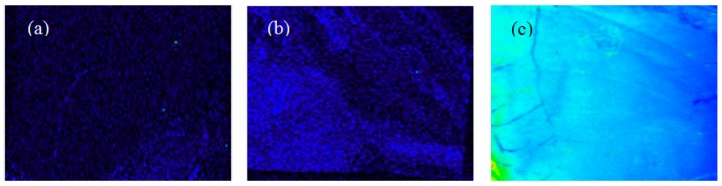
Fluorescence images of the binding of fluorescently labeled aptamers to Au nanostructures with/without chitosan coating. Quenching effect vs. distance between Au and dye was investigated with the fluorescent aptamers absorbed to the substrate of (**a**) Au deposited by E-beam evaporator without chitosan coating; (**b**) Au with needle-like structures without chitosan coating; and (**c**) Au with needle-like structure coated with chitosan (1000 Da).

It is clearly found that in the presence of Au nanostructure the fluorescent intensity is considerably enhanced relative to that without the Au nanostructure (Figure 4a–c). Fluorescence signals of Cy3 both on bare Au nanostructure are shown in Figure 4b and chitosan-coated Au nanostructure in Figure 4c (λ = 585 nm). In the presence of Au nanostructure and chitosan coating, the fluorescence intensity is strongest throughout the whole range of substrates. The positively charged chitosan layer on nano Au structure facilitated DNA binding. The uniform fluorescence measurement was achieved that confirmed the uniform large-area synthesis of Au nanostructures and chitosan coating. Chitosan could be simply coated through their electrostatic affinity towards Au surface. Its uniformity was validated with the image of fluorescence in 5 mm width as in Figure 4. The uniform fluorescence of conjugated DNAs represents the reliable coating of the positively charged chitosan layer on Au substrates.

### 3.3. Enhancement of Fluorescence on Au Substrate

In the presence of gold nanostructures the fluorescent intensity is increased with respect to effective surface area. In addition, the fluorescence might be enhanced from surface plasmon of metallic nanostructure. The factors that affect enhancement of surface plasmon are the characteristics of the material and the structure. The surface enhancement of both bare gold and gold nanostructures was comparatively investigated as in Table 1. If the material and structure are identical, the distance of fluorophore and metal is a critical parameter for surface enhancement. When the distance between metal surface and dye is maintained 0–20 nm, the quenching effect is dominant. When the gap increases, fluorescence from dyes was less quenched by Au. For the enhancement of fluorescence, quenching effect needs to be avoided by maintaining the distance greater than 20 nm. However, the distal location is not favored since the surface plasmon diminished rapidly with the gap. Thus, optimization of the distance between fluorophore and metal was controlled with chitosan layer. With chitosan of molecular weights 1000 and 5000 Da, the effect of distance was comparatively investigated. For the third parameter of surface enhancement, the fluorescent dyes of different quantum yield were compared, namely FAM and Cy3 dye. In Table 1, ratio of fluorescence intensity between the substrate of Au nanostructures and the one of bare Au were listed, i.e., I_n_/I_b_. For the dye of high quantum yield, FAM, the enhancement from nanostructure was about 2.6. The effect was almost irrelevant to the chitosan coating that modulates the gap between the fluorophore and metal. The 2.6 times enhancement of fluorescence is ascribed to the increase of effective surface area that is independent from the gap. In addition, FAM has very high quantum yield and is hardly enhanced with surface plasmon as previously described [29].

On the contrary, the effect of chitosan coating is distinct with the dye of low quantum yield, Cy3. The unique signal enhancement from low quantum yield dye, Cy3 coated onto thin chitosan layer from low molecular weight chitosan (1000 Da) could be explained from the surface enhancement on metal nanostructures. The chitosan layer of low molecular weight is assumed to generate proper distance between Cy3 and Au, avoiding quenching and still capable of surface plasmon enhancement. Fluorescence enhancement from Cy3-conjugated DNA on thicker chitosan layer was slightly stronger than the one from FAM-conjugated DNA. This could also be ascribed to the surface enhancement but the effect was minor due to the larger separation of fluorophores from metal surface. In all, it conforms with the report that the dyes of low quantum yield could be enhanced more favorably from the surface plasmon.

In order to obtain the benefits of surface plasmon on Au nanostructures, the substrate was prepared with chitosan coating of molecular weight of 1000 Da and the fluorophore was determined to be Cy3 for the following assays.

### 3.4. Injection of Thrombin and Quantitative Measurement of Fluorescence

#### 3.4.1. In Situ Fluorescence upon Thrombin Injection

**Figure 5:**
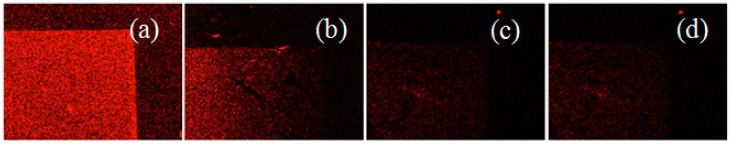
In situ Fluorescent signal measurements after thrombin (10 μM) injection at (**a**) 0 min (**b**) 4.5 min (**c**) 9 min and (**d**) 15 min.

Based on the optimized parameters for the surface plasmon, one-step assay (Figure 1) was carried out with the thrombin specific aptamer [33]. The fluorescent aptamers were immobilized on the chitosan-coated substrates and 1% BSA was added to reduce non-specific response after target injection. Upon addition of thrombin to the sensing zone of the substrate, proteins bind to the aptamers and constitute complexes of protein-aptamers. The negative charge density fastening aptamers to the chitosan layer became much weaker and the protein–aptamer complexes were released from the surface of nanostructures. As a result of the separation process, fluorescence intensity from the substrate decreased. Real-time detection of fluorescence after injection of thrombin was captured in Figure 5a–d.

Fluorescence from the nanostructure substrate decreased gradually for about 10 min and stabilized. The fade-off of the fluorescence could be explained by two hypotheses. When the aptamer settled down to the binding pocket of target protein, thereby constructing a complex, its negative charge was partially shielded with the protein. As the fluorescent aptamer comprises the protein–aptamer complex and escaped from the surface, the fluorescent dye was getting far from the metallic surface, then the surface plasmon enhancement disappeared. Another possible explanation is the conformation change of aptamer induced from the target protein binding. If the fluorescent terminal comes close enough to the surface, the fluorescence might be quenched by Au nanostructure. Either of the two mechanisms enables the signal change, i.e., decrease of fluorescence intensity, without additional washing step of fluorescent aptamer. As irrelevant proteins or DNA sequences with similar size were adopted, negligible fluorescence change was observed (Data not shown). The fluorescence change occurred selectively for the specific aptamer–target binding.

#### 3.4.2. Effect of the Length of Aptamers

**Figure 6:**
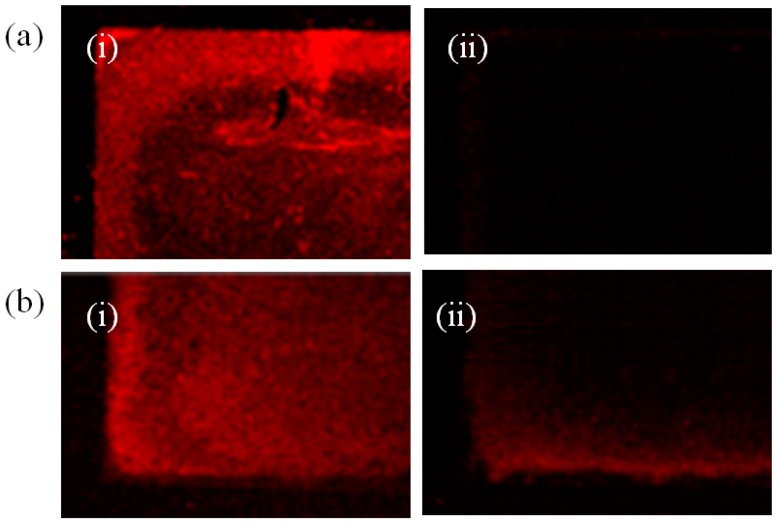
Fluorescence signal measurements with respect to different aptamer lengths (**a**) 15mer (**b**) 21mer of thrombin specific aptamers (**i**) just after thrombin solution was added (**ii**) after 15 min incubation and rinse.

The effect of strength of electrostatic binding between the chitosan and aptamer was investigated by varying the stem length of thrombin-binding aptamer. The change in fluorescence intensity with respect to length of thrombin-binding aptamer was measured with 15mer and 21mer. Thus prepared DNA aptamer comprises the core thrombin-binding sequence of 5’-GGT TGG GGT TGG-3’ and a 5’-extended thrombin-binding DNA aptamer [21mer: 5’-CAC TGT GGT TGG TGT GGT TGG-3’]. A 10 μg/mL thrombin in binding buffer was injected onto the fabricated gold nanostructures. After reaction with the fabricated gold nanostructures, it was observed that the bright red area turned dark. Figure 6a,b shows the change in fluorescence intensity after thrombin injection in both short and long aptamers case. The final measurements were performed after brisk rinse with binding buffer following at 15 min incubation with thrombin solution.

It was found that the 15mer reduced the fluorescence intensity 20% more than 21mer proving that shorter aptamer stem length is advantageous for sensitive measurements. As described above, there is a chance of distinct conformation change of aptamer upon protein binding leading to a change in the distance between aptamer and Au nanostructure. This in turn might induce quenching of fluorescence. In this case, the additional length of aptamer irrelevant to the protein binding should not affect the fluorescent signal. However, if the fluorescent aptamers were departed from the nanostructure due to the weakening of the electrostatic attraction, strong negative charge of longer aptamer might keep the aptamers attracted to the chitosan layer. Based on the increased signal change for short aptamers, charge shield and departure mechanism seemed more convincing than conformational change theory. Presumably, the 6 bases other than the 15 base-protein binding region kept the electrostatic interaction with chitosan layer even after protein binding, more aptamers remained bound on the nanostructure and the fluorescence did not change as much as shorter DNA case.

#### 3.4.3. Thrombin Assay

**Figure 7:**
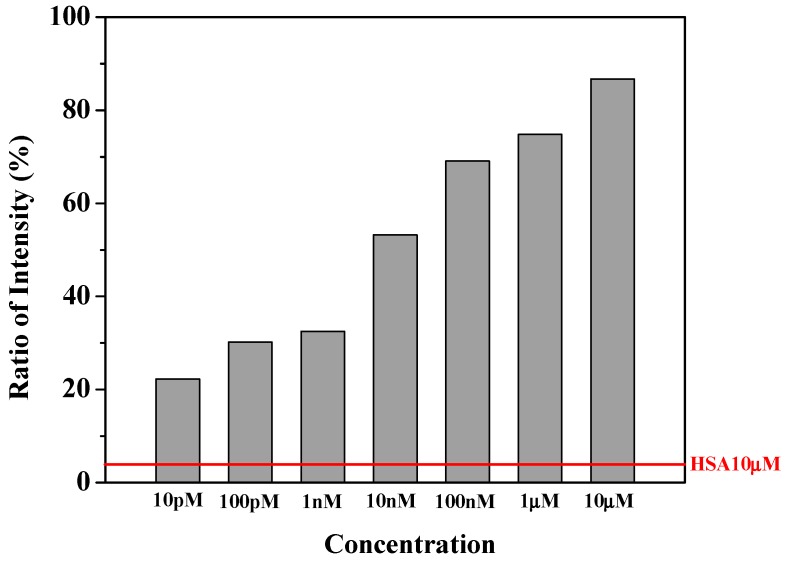
Graph representing fluorescence intensity in relation to concentration of targets of thrombin and human serum albumin (HSA).

The change in fluorescence intensity was studied with respect to the change in concentration of thrombin. The reaction time of 15 min is kept as a constant as in the previous measurements. The concentration of thrombin was varied as 10 μM, 1 μM, 100 nM, 10 nM, 1 nM, 100 pM and 10 pM and it was observed that there was a decrease in fluorescence intensity respectively. Figure 7 shows the quantitative analysis of thrombin using the nanostructured aptamer-based sensors. The fluorescence changed proportional to the concentration of the target protein in a very wide dynamic range say 6 orders of magnitudes. The non-specific protein could interfere with the electrical environment of aptamers, thereby weakening the aptamers binding to chitosan layer. Human serum albumin (HSA) has a lower isoelectric point (pl = 5.3) than that of 7.4 buffer pH, leading to a net negative charge. The false-positive signal might be resulted from the aptamer replacement by negatively charged HSA. However, the fluorescence decreased less than 5% at the concentration of 10 μM of HSA producing weaker signal than 10 pM of thrombin. In fact, the sensor was not only sensitive but also selectively responding.

## 4. Conclusions

A simple and selective single-step aptamer-based surface-enhanced fluorescent optical sensor was demonstrated by aptamer conjugation with Cy3 immobilized on the nanogold substrate through electrostatic attraction. A new nanogold substrate on copper thin film was constructed through a simple and cost-effective electroless plating methodology. The gold substrate was coated with chitosan and the optimum molecular weight of chitosan is identified. The developed sensor enabled fast, selective detection and quantization to the sub-nM level of target protein (thrombin). The fabrication of gold nanostructures allows the enhancement of fluorescence by plasmon effect, thereby increasing the sensitivity of detection. The detection of molecules in real time is also possible. The fabricated gold nanostructures play a vital role as a sensor for molecules involved in protein/DNA binding which can be extensively applied to various reliable aptamer/target pairs. Moreover, the ability of aptasensor to detect protein concentrations at picomolar level may be further extended for a robust optical sensor that performs detection in real time without rinse.

## Figures and Tables

**Table 1:** Fluorescence measurement for both 1000 and 5000 Da chitosan-coated Au nanostructures using FAM and Cy3 dyes.

Chitosan	I_n_/I_b_ FAM (Q.Y: 90%)	I_n_/I_b_ Cy3 (Q.Y: 10%)
With “M.W. = 5000“	2.64 ± 0.08	2.98 ± 0.90
With “M.W. = 1000“	2.58 ± 0.09	7.62 ± 0.60
